# Eye movement desensitization and reprocessing (EMDR) therapy for posttraumatic stress disorder in adults with serious mental illness within forensic and rehabilitation services: a study protocol for a randomized controlled trial

**DOI:** 10.1186/s13063-019-3760-2

**Published:** 2019-11-21

**Authors:** Susanna Every-Palmer, Tom Flewett, Shaystah Dean, Oliver Hansby, Atalie Colman, Mark Weatherall, Elliot Bell

**Affiliations:** 1 University of Otago, Wellington, New Zealand; 2 Capital and Coast District Health Board, Wellington, New Zealand

**Keywords:** Eye movement desensitization and reprocessing (EMDR) therapy, Mental health, Forensic psychiatry, Posttraumatic stress disorder, Rehabilitation, Trauma, Offending

## Abstract

**Background:**

Eye movement desensitization and reprocessing (EMDR) is an evidenced-based treatment for posttraumatic stress disorder (PTSD). Forensic mental health services provide assessment and treatment of people with mental illness and a history of criminal offending, or those who are at risk of offending. Forensic mental health services include high, medium, and low-security inpatient settings as well as prison in-reach and community outpatient services. There is a high prevalence of PTSD in forensic settings and posttraumatic experiences can arise in people who violently offend in the context of serious mental illness (SMI). Successful treatment of PTSD may reduce the risk of relapse and improve clinical outcomes for this population. This study aims to assess the efficacy, risk of harm, and acceptability of EMDR within forensic and rehabilitation mental health services, as compared to treatment as usual (routine care).

**Methods:**

This is a single-blind, randomized controlled trial comparing EMDR therapy to the waiting list (routine care). Adult forensic mental health service users (*n* = 46) with SMI and meeting the criteria for PTSD will be included in the study. Participants will be randomized after baseline assessment to either treatment as usual plus waiting list for EMDR or to treatment as usual plus EMDR. The EMDR condition comprises nine sessions, around 60 min in length delivered weekly, the first of which is a case conceptualization session. The primary outcomes are clinician and participant-rated symptoms of PTSD, and adverse events. Secondary outcomes include psychotic symptoms, social functioning, level of disability, self-esteem, depressive symptoms, post-trauma cognitions, and broad domains of complex posttraumatic difficulties. A trained assessor blinded to the treatment condition will assess outcomes at baseline, 10 weeks, and 6 months. Additionally, grounded theory qualitative methods will be used to explore participant experience of EMDR for a subset of participants.

**Discussion:**

This study will contribute to the currently limited evidence base for EMDR for PTSD in forensic settings. It is the first randomized clinical trial to assess the efficacy, risk of harm, and acceptability of EMDR for PTSD in people with SMI in either forensic, mental health inpatient, or custodial settings.

**Trial registration:**

Australia and New Zealand Clinical Trials Network, ACTRN12618000683235. Registered prospectively on 24 April 2018.

## Background

Posttraumatic stress disorder (PTSD) in forensic populations is common, disabling, and often unaddressed [1–7]. There is converging evidence that forensic patients often have extensive trauma histories [8] and that their trauma syndromes, which frequently present with comorbid serious mental illness (SMI), typically schizophrenia spectrum conditions and severe mood disorders [9], in turn can be associated with offending [10].

The symptoms of PTSD include: ongoing re-experiencing of an event or events involving death, or actual or threatened serious injury or violence (e.g., through nightmares or distressing memories); avoiding reminders of the trauma (e.g., places or people associated with it); negative thoughts or feelings pertaining to the trauma (e.g., self-blame or difficulty remembering aspects of what happened); and, finally, physical arousal or reactivity (e.g., startling easily or difficulty sleeping). A formal diagnosis is made when the symptoms are so severe that the sufferer is not able to function adequately in one or more aspects of their life (e.g., at work or in social relationships) [11].

Eye movement desensitization and reprocessing (EMDR) is a talking therapy that was developed for the treatment of posttraumatic states including PTSD [12]. It is now well established as an effective treatment with a strong evidence base [13, 14] and is recommended by the National Institute for Health and Care Excellence (NICE) guidelines and by the World Health Organization for the treatment of PTSD [15]. During EMDR the client is instructed to think about the traumatic event for brief periods while simultaneously tracking the therapist’s fingers (which are moved from side to side) with their eyes. The resulting bilateral brain stimulation is thought to activate the brain’s information processing pathways, enabling more adaptive associations to be made. Over time, this leads to processing of traumatic events into long-term memory. As a result, people become desensitized to the trauma, allowing their symptoms to remit, and their functioning in life to improve [15].

Promisingly, a large randomized controlled trial (RCT) conducted by van den Berg et al. [16] found EMDR of significant benefit in community patients with serious mental illness and PTSD, showing improvement in both posttraumatic stress symptoms and the symptoms of comorbid mental illnesses, including psychotic disorders. Adverse events and rates of re-victimization were lower in the EMDR group than in the control group.

Despite strong evidence for high rates of PTSD in forensic populations [1], there are no trials testing EMDR compared with a control group in the forensic setting. There are, however, favorable case reports of EMDR being used in forensic settings, with at least six case studies demonstrating reductions in offence-related PTSD symptoms [17–22].

Untreated PTSD in forensic services has costs to the individual, their families, and society; costs which may be exacerbated where PTSD presents concurrently with other serious SMI. PTSD may serve as a chronic stressor, worsening symptoms of other mental conditions, and/or predisposing people to substance misuse, further compromising their recovery.

EMDR has the potential to be of benefit to the forensic mental health population. The intervention is particularly suited to people in secure care because it can take place anywhere, and for people with SMI because it does not require homework. The desensitization in EMDR is limited to imaginal activities and does not involve the real-world (in vivo) graduated exposure to trauma triggers generally required in other evidence-based PTSD therapies.

This trial builds on the work of van den Berg et al.’s positive findings in RCTs examining EMDR for PSTD in people with psychotic illnesses [23–25], extending this to samples in forensic inpatient, outpatient, and prison services who have the additional complex overlay of criminal offending issues.

## Study aims

### Primary aim

In a sample of people receiving forensic and rehabilitation mental health care, we aim to examine: the efficacy of EMDR versus the waiting list for treating PTSD symptomology; and the safety and acceptability of EMDR versus the waiting list as indicated by adverse events.

### Secondary aims

Other aims are to assess the impact of EMDR versus the waiting list on psychotic symptoms, social functioning, self-esteem, and depressive symptoms. Additionally, this study aims to gain in-depth insight into the subjective experience of EMDR for consumers of forensic mental health services.

## Methods/design

### Study design

This study will be a single-blind, randomized, assessor-blinded, controlled trial designed in accordance with the Consolidated Standards of Reporting of Trials (CONSORT) guidelines. It is a superiority design and is not a crossover trial.

Participants will be assigned to EMDR therapy or waiting list by independent randomization and outcomes will be assessed using well-validated measures with robust psychometric properties. The trial will be conducted at the Central Regional Forensic and Rehabilitation Mental Health Service in New Zealand. The allocation sequence will be determined by the date the patients are referred, and will be concealed from those assigning participants to intervention groups. A qualitative adjunct to explore treatment acceptability is included in this study.

**Fig. 1:**
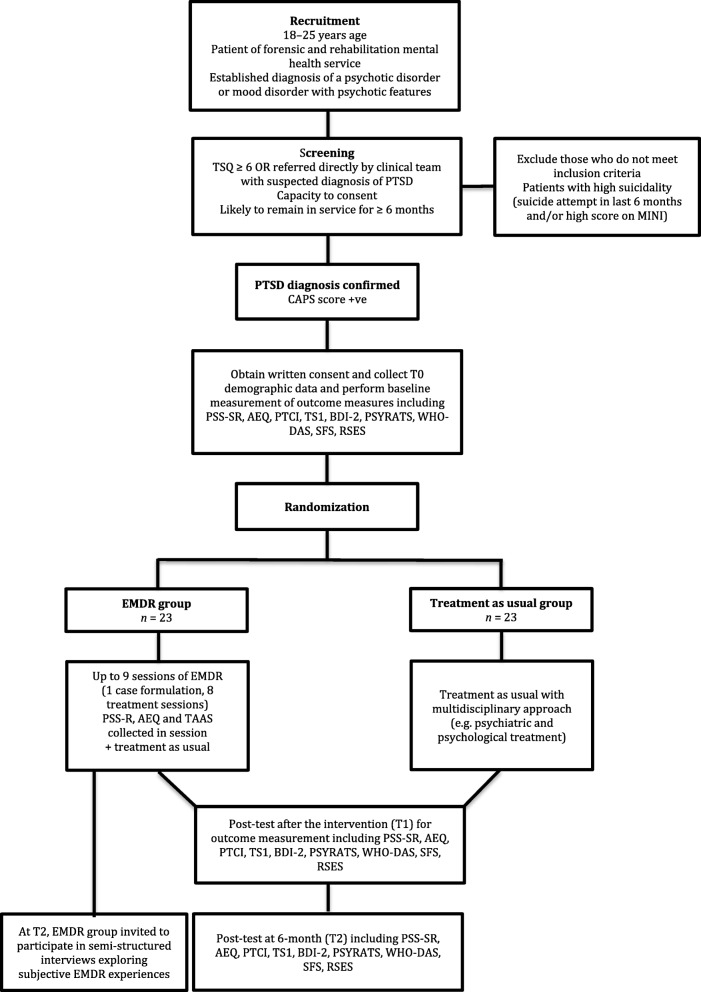
Consolidated Standards of Reporting Trials (CONSORT) flow diagram. AEQ, Adverse Events Questionnaire; BDI-II, Beck Depression Inventory—second edition; CAPS, Clinician Administered PTSD Scale; EMDR, eye movement desensitization and reprocessing; MINI, Mini International Neuropsychiatric Interview; PSS-SR, Posttraumatic Stress Disorder Symptom Scale—Self-Report; PSYRATS, Psychotic Symptom Rating Scales; PTCI, Posttraumatic Cognitions Inventory; RSES, Rosenberg Self-Esteem Scale; SFS, Social Functioning Scale; TAAS, Treatment Acceptability/Adherence Scale; TSI-2, Trauma Symptom Inventory—second edition; TSQ, Traumatic Symptom Questionnaire; WHODAS, World Health Organization Disability Assessment Scale

This protocol is presented in accordance with the Standard Protocol Items: Recommendations for Interventional Trials (SPIRIT) 2013 statement (see Additional file 1) and the SPIRIT figure (Fig. 1).

### Ethical approval

The study has received full ethics approval from the New Zealand Health and Disability Ethics Committee (HDEC, reference 18/CEN/48), and has been subject to Māori consultation^1^ with the Ngāi Tahu Research Committee (10 April 2018).

### Participants

Participants will be recruited from the forensic and rehabilitation mental health service from May 2018 until November 2020 (approximately). Participants will be recruited from inpatient, custodial, and community settings and must all be current patients of the forensic and rehabilitation service.

The eligibility criteria are as follows:

- Age between 18 and 65 years
- A lifetime history of a psychotic disorder or a mood disorder with psychotic features diagnosed according to ICD-10 or DSM-5 criteria
- Meeting diagnostic criteria for PTSD (Clinician Administered PTSD Scale (CAPS))
- Current patient of the forensic and rehabilitation mental health services
- Competent to provide informed consent
- Likely to remain in the area for the 6-month trial duration (i.e., if a prisoner, release date at least 6 months away; if a patient within a forensic mental health service, their anticipated discharge is at least 6 months away)

To ensure “real world” generalizability of study results, we are keeping exclusion criteria to a minimum. The exclusion criteria are as follows:

- High suicidality, operationalized as the combination of having a high suicidality score on the Mini International Neuropsychiatric Interview (MINI-plus) or with a suicide attempt within the past 6 months
- Mental state considered by the treating psychiatrist as too unstable to participate in the trial (participants may be experiencing chronic low-grade symptoms, but cannot be acutely psychotic)
- Insufficient competence in the English language

The only intervention that is not permitted during the trial is the administration of EMDR by an external treatment provider. If participants intend to access EMDR through a private provider or Accident Compensation Corporation services (New Zealand’s no-fault accidental injury scheme) during the trial period, they will be ineligible for inclusion. Other interventions (e.g., psychological therapy, medication) are permitted.

### Recruitment

As part of the standard forensic care contacts, people who clinicians consider may fulfill the study criteria (i.e., have a serious mental illness, possible PTSD, and are competent to consent) will be given an information sheet about the project and invited to complete the Trauma Screening Questionnaire (TSQ) [26] by their clinician. The TSQ is a screening test for PTSD which has demonstrated adequate sensitivity and specificity, and good criterion validity [27]. If participants have a TSQ score ≥ 6 (or ≥ 5 for the subset of participants taking therapeutic doses of marked or moderately sedating antipsychotics, specifically clozapine ≥ 250 mg/day, olanzapine ≥ 15 mg/day, or quetiapine ≥ 300 mg/day [28], as this group experiences excessive sleep [29] and for whom we will exclude the disrupted sleep question) they will be eligible for formal PTSD assessment. Potential participants who verbally consent to receive more information about the study will be contacted by one of the investigators (either by telephone or if they do not have a telephone, for example if they are in custodial care, then through their clinical team) to arrange a face-to-face meeting. Patients can also be referred directly by their treating teams for an inclusion interview if they have a clinical diagnosis of PTSD.

At the face-to-face meeting, more detailed verbal information about the study will be provided by one of the investigators and the potential participant will have the opportunity to ask further questions about the study. Screening for suicidality will also take place at this stage (as previously described). People at high risk for suicidality will be excluded.

If the person decides to participate, the investigator will obtain written informed consent after explaining the purpose and content of the study, rights of participants, and confidentiality. An appointment will be made for confirmation of a PTSD diagnosis.

At the inclusion assessment, the researchers will administer the Clinician Administered PTSD Scale (CAPS) [30] structured interview to determine both eligibility for the trial and also the severity of symptoms at baseline. A positive CAPS is considered the gold standard in determining the presence of diagnoses of PTSD [31], with strong retest and inter-rater reliability, internal consistency, convergent and discriminant validity, diagnostic utility, and sensitivity to change [32].

Eligible patients will provide informed consent and complete the baseline assessments.

The outcome measures are assessed at baseline (T0), again following treatment at 2 months (T1), and then 6 months (26 weeks) after treatment started (T2). The outcome measures are described in detail in the following pages.

### Randomization, allocation concealment, and blinding

Consenting participants will receive the pre-treatment (T0) measurements. After T0, participants will be randomly assigned to either the EMDR or treatment as usual group. An independent research manager at the District Health Board research office (who is not involved in the study) will generate the random sequence. This allocation sequence will be computer-generated and non-stratified with varying block sizes, which will not be disclosed to the researchers to ensure concealment. After randomization, the research office will notify the trial coordinator from the research team as to the allocation. To ensure allocation concealment, the randomization code will not be released until after the participant has been recruited into the trial, which takes place after all baseline measurements have been completed.

After allocation, the participants will be informed which group they are in. Hence, neither the participants nor the specialized therapists who deliver EMDR will be blinded to the allocation (as it is not practical to undertake blinded EMDR). However, outcome assessors will be blinded to treatment allocation, as will those performing data entry and the trial statistician (MW). Assessors and therapists will emphasize the importance of blinding to the participants and remind them not to reveal the randomized treatment condition. Assessors will avoid contact with the therapists and other caregivers. Assessors will be asked to report any case of unblinding, in which case, another assessor will repeat the entire measurement.

### Therapist training requirements

The treatment sessions will be delivered by consultant psychiatrists and clinical psychologists who have completed at least basic training in in an EMDR International Association (EMDRIA)-recognized program and have experience working with people with SMI. The exact criteria are as follows.

All EMDR therapists must:

- Be practicing mental health clinicians with full registration to practice as clinical psychologists or psychiatrists, with a current practicing certificate issued by the New Zealand Psychologists Board (for psychologists) or by the New Zealand Medical Council (for psychiatrists)
- Have at least 5 years of experience working with people with serious mental illness
- Have completed both EMDR Basic Training Stage 1 and Stage 2 (as a minimum) in a training program recognized by the EMDRIA

Therapists will be supervised by a senior professional certified in EMDR.

Ten percent of sessions will be observed and reviewed for treatment fidelity using the EMDR Fidelity Rating Scale [33], which evaluates adherence to EMDR’s eight-phase/three-pronged protocol.

### Intervention

#### Choice of comparators

While the importance of trauma is increasingly being recognized [1–10], New Zealand forensic mental health services do not systematically screen for PTSD or routinely offer trauma-focused psychological therapy (such as EMDR or trauma-focused cognitive behavioral therapy (CBT)). There is no trial-based evidence investigating whether EMDR in this group is effective. This trial is a superiority trial which investigates whether treatment with EMDR improves outcomes in reducing PTSD symptomatology (alongside other important outcomes described in the following) when added to treatment as usual, compared to treatment as usual alone.

#### Treatment as usual

Participants in both groups—EMDR and waiting list—receive treatment as usual, consisting of antipsychotic medication and multidisciplinary treatment by psychiatrists, psychologists, social workers, occupational therapists, key workers, and cultural workers. Psychotic illness will be treated by a multidisciplinary approach in accordance with the Royal Australian and New Zealand College of Psychiatrists clinical practice guidelines for the management of schizophrenia and related disorders [34]. Treatment as usual will be considered equal in both conditions as a result of the randomization procedure and participants receiving similar care within the same service.

#### Experimental intervention: EMDR

In addition to treatment as usual, the participants randomized to EMDR will undergo nine sessions of EMDR therapy, each 60-min long. The first session will involve assessment and planning, and the subsequent eight sessions will involve active EMDR treatment.

In the first session, the treatment rationale will be explained. The therapist will receive information from the Interview for Traumatic Events in Childhood (ITEC), a reliable and valid tool for assessing early traumas [35]. The ITEC captures categories (e.g., sexual abuse, physical abuse) of experienced traumatic events. In addition, the intensity of re-experiencing symptoms of each traumatic event is assessed. Based on the ITEC, the therapist draws up a preliminary, formatted EMDR case conceptualization (when crucial memories are identified for reprocessing [36]), which they discuss and complete together with the patient in the first session. This results in a hierarchy of the most relevant traumatic memories in order of significance with regard to the symptoms of PTSD.

During the next eight sessions, the identified traumatic memories will be processed following the basic EMDR protocol. The participant is asked to focus on the currently most distressing image of a memory in a multimodal manner, including its image, thought (an experienced negative cognition (NC) and a healthier positive cognition (PC)), emotion, physical sensation, and level of tension. The patient then rates this memory on a subjective unit of distress (SUD) scale from 0 to 100. Processing of the memory starts when the therapist asks the patient to hold the target image in mind while concentrating on a distracting stimulus (the finger of the therapist eliciting eye movements) for about 30 s. The patient reports briefly what comes to mind and is guided by the clinician to refocus on that element while focusing on the distracting stimulus. This continues until no more associations come up and the disturbance level associated with the target memory (SUD) drops to zero.

The therapist then guides the participant in installing the PC to a maximum validity, that is, a Validity of Cognition (VOC) scale score of 7 (a “completely true” rating). The participant identifies any residual disturbing sensations and, if present, the physical disturbance is processed in the same manner as already described. The therapist facilitates a positive closure to the session. In the next session, a re-evaluation takes place in which the participant comments on previously processed targets as a basis for further intervention.

#### Early completers

We will allow “early completion” when symptoms resolve before the nine sessions are finished. A participant will be considered an early completer of treatment when his or her score on the PTSD Symptoms Scale—Self Report (PSS-SR) is lower than 10 on two consecutive occasions, and if the SUD scores from all situations that are part of the case conceptualization are reduced to zero. If this happens, therapy can be stopped early.

### Measures

#### Demographic data

Demographic data collected will include age, gender, ethnicity, mental health diagnoses, legal status, and index offence (the offence/s for which they are currently detained) details, including date and type, relationship of the victim, and intoxication at the time of the event.

#### Outcome measures

**Fig. 2:**
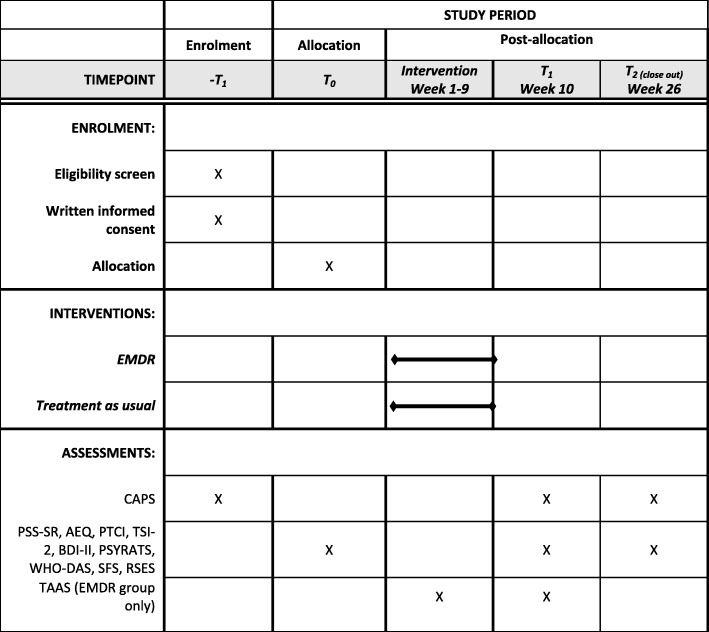
Standard Protocol Items: Recommendations for Interventional Trials (SPIRIT) figure. AEQ, Adverse Events Questionnaire; BDI-II, Beck Depression Inventory—second edition; CAPS, Clinician Administered PTSD Scale; EMDR, eye movement desensitization and reprocessing; PSS-SR, Posttraumatic Stress Disorder Symptom Scale—Self-Report; PTCI, Posttraumatic Cognitions Inventory; PSYRATS, Psychotic Symptom Rating Scales; RSES, Rosenberg Self-Esteem Scale; SFS, Social Functioning Scale; TAAS, Treatment Acceptability/Adherence Scale; TSI-2, Trauma Symptom Inventory—second edition; WHODAS, World Health Organization Disability Assessment Scale

A range of primary, secondary, and tertiary outcome measures (all outlined in the following, see Fig. 2) will be administered at baseline (T0), post treatment (T1, at 10 weeks), and at 6-month follow-up (T2). After T2, participants will be invited to engage in a semi-structured interview (see Subjective experiences of EMDR: qualitative data collection) to gather data on their subjective experience of PTSD and EMDR.

T2 is the starting point for treatment for the waiting list group. For participants in the EMDR group, additional treatment-related assessments take place within sessions (see later).

#### Primary outcome measurements: PTSD symptoms, adverse events, and treatment acceptability

There are two primary outcome measures pertaining to PTSD symptoms, two regarding treatment safety, and one measure of the acceptability of EMDR treatment. The first primary outcome measure is the CAPS, which is a 30-item structured interview that assesses DSM-5 PTSD symptoms [37]. The CAPS will be used to assess the presence or absence of PTSD diagnosis and the frequency and intensity of the clinician-rated PTSD symptoms.

**Table 1:** Secondary outcome measures

Secondary outcome	Measurement instrument^a^	T0	T1	T2
Posttraumatic cognitions	PTCI (s)	X	X	X
Self-disturbance	TSI-2 Factor 1 (s)	X	X	X
Posttraumatic stress	TSI-2 Factor 2 (s)	X	X	X
Externalization	TSI-2 Factor 3 (s)	X	X	X
Somatization	TSI-2 Factor 4 (s)	X	X	X
Depression	BDI-II (s)	X	X	X
Psychosis	PSYRATS (i)	X	X	X
Overall functioning	WHODAS 2.0 (i)	X	X	X
Social functioning	Social Functioning Scale (i)	X	X	X
Self-esteem	RSES (s)	X	X	X

^a^ (i) = measurement interview, (s) = self-report

BDI-II, Beck Depression Inventory—second edition; PTCI, Posttraumatic Cognitions Inventory; PSYRATS, Psychotic Symptom Rating Scales; RSES, Rosenberg Self-Esteem Scale; T0, baseline; T1, post treatment; T2, 6-month follow-up; TSI-2, Trauma Symptom Inventory—second edition; WHODAS, World Health Organization Disability Assessment Scale

**Table 2:** Items from the World Health Organization Trial Registration Data Set

Data category	Information
Primary registry and trial identifying number	Australia and New Zealand Clinical Trials Network, registration number ACTRN12618000683235 (registered prospectively)
Date of registration in primary registry	26 April 2018
Sponsor	University of Otago Contact details: Office of Research and Enterprise, Otago University, Level 1, 87 St David Street, Dunedin, New Zealand. Tel. + 64 34 798 905
Contact for public/scientific queries	susanna.every-palmer@otago.ac.nz
Public title	Eye movement desensitization and reprocessing (EMDR) therapy for posttraumatic stress disorder in adults with serious mental illness within forensic and rehabilitation services: a study protocol for a randomized controlled trial
Scientific title	Eye movement desensitization and reprocessing (EMDR) therapy for posttraumatic stress disorder in adults with serious mental illness within forensic and rehabilitation services: a study protocol for a randomized controlled trial
Countries of recruitment	New Zealand
Health condition(s) or problem(s) studied	PTSD in people receiving care from forensic mental health services
Intervention(s)	EMDR therapy
Key inclusion and exclusion criteria	Inclusion criteria: aged 18–65 years; a lifetime history of a psychotic disorder or a mood disorder with psychotic features as diagnosed according to ICD-10 or DSM-5 criteria; meeting diagnostic criteria for PTSD (Clinician Administered PTSD Scale (CAPS)); current patient of the forensic and rehabilitation mental health services; competent to provide informed consent; likely to remain in the area for the 6-month trial duration (i.e., if a prisoner, release date at least 6 months away; if a patient within a forensic mental health service, their anticipated discharge is at least 6 months away) Exclusion criteria: high suicidality, operationalized as the combination of having a high suicidality score on the Mini International Neuropsychiatric Interview (MINI-plus) or with a suicide attempt within the past 6 months; mental state considered by the treating psychiatrist as too unstable to participate in the trial (participants may be experiencing chronic low-grade symptoms, but cannot be acutely psychotic); insufficient competence in the English language to understand, provide informed consent, and participate in therapy and data collection
Study type	Single-blind, randomized controlled trial
Date of first enrolment	25 May 2018
Target sample size	46
Recruitment status	Currently recruiting
Primary outcome(s)	Change in PTSD symptoms (measured by the CAPS and PSS-SR), treatment safety (measured by the adverse event questionnaire), and acceptability of the EMDR treatment (measured by the TAAS and by qualitative participant feedback)
Key secondary outcomes	Posttraumatic cognitions (PTCI), self-disturbance (TSI-2 Factor 1), posttraumatic stress (TSI-2 Factor 2), externalization (TSI-2 Factor 3), somatization (TSI-2 Factor 4), depression (BDI-II), psychosis (PSYRATS), overall functioning (WHODAS 2.0), social functioning (Social Functioning scale), self-esteem (RSES)

BDI-II, Beck Depression Inventory—second edition; DSM-5, *Diagnostic and Statistical Manual of Mental Disorders*, fifth edition; EMDR, eye movement desensitization and reprocessing; ICD-10, International Classification of Diseases, 10th edition; PSS-SR, Posttraumatic Stress Disorder Symptom Scale—Self-Report; PTCI, Posttraumatic Cognitions Inventory; PSYRATS, Psychotic Symptom Rating Scales; PTSD, posttraumatic stress disorder; RSES, Rosenberg Self-Esteem Scale, TAAS, Treatment Acceptability/Adherence Scale; TSI-2, Trauma Symptom Inventory—second edition; WHODAS, World Health Organization Disability Assessment Scale

We will consider the CAPS to provide a diagnosis of PTSD if the person scores positively for all of the following conditions [11]:

- At least one of the Criterion B symptoms (“re-experiencing” symptoms, relating to diagnostic items 1–5 in the CAPS)
- At least one of the Criterion C symptoms (“avoidance” symptoms, relating to diagnostic items 6–7 in the CAPS)
- At least two of the Criterion D symptoms (“negative alterations in cognition and mood”, relating to diagnostic items 8–14 in the CAPS)
- At least two of the Criterion E symptoms (“hyperarousal” symptoms, relating to diagnostic items 15–20 in the CAPS) OR at least one Criterion E symptom for the subset of participants taking therapeutic doses of marked or moderately sedating antipsychotics (specifically, clozapine ≥ 250 mg/day, olanzapine ≥ 15 mg/day, or quetiapine ≥ 300 mg/day [28])
  AND
- Criterion F is met (disturbance has lasted 1 month)
- Criterion G is met (disturbance causes either clinically significant distress or functional impairment)

The CAPS is considered the gold standard to diagnose posttraumatic stress disorder and to establish its severity. A review of the empirical literature on psychometric properties of the CAPS [32] found that the CAPS has excellent reliability (> 0.90), yielding consistent scores across items, raters, and testing occasions. There is also strong evidence of validity with excellent (> 0.90) convergent and discriminant validity, diagnostic utility, and sensitivity to clinical change.

Secondly, the Posttraumatic Stress Disorder Symptom Scale—Self-Report (PSS-SR) [38] will be administered to assess self-reported severity of PTSD symptoms [39]. The PSS-SR consists of 17 items corresponding to the 17 diagnostic DSM-IV-TR criteria of PTSD which are rated on a 3-point Likert scale, where 0 = *not at all*, 1 = *a little* bit, 2 = *somewhat*, and 3 = *very much*. This yields a total score measuring symptom severity (range 0–51), as well as separate severity scores for re-experiencing (range 0–15), avoidance (range 0–21), and arousal (range 0–15). The PSS-SR will also be administered at T0, T1, and T2, as well as before each treatment session, to assess changes in the PTSD symptoms during treatment. The PSS-SR has revealed satisfactory internal consistency (Cronbach’s α = 0.91), high test–retest reliability (*r* = 0.74), and good concurrent validity (sensitivity = 62%, positive predictive power = 100%, negative predictive power = 82% [38, 40]).

Two primary outcome measures pertain to adverse events. The Adverse Events Questionnaire (AEQ), which we have designed using similar questions to Van den Berg et al. [16], is a checklist that will help establish treatment safety. In 10 questions, the patient is asked to report any experiences they have had over the last 2 months of self-harm, suicide attempt, deliberately hurting another person, excessive use of alcohol, excessive use of drugs, having needed help because of a crisis, moving back to a higher level of security within the services, or any reoffending.

The Adverse Events Session Rating scale will also be used to assess adverse events in the participant before and after treatment sessions (sessions two and three), for example, being suicidal and hearing voices. Participants will respond on a 10-point visual analog scale (from “*no, not at all*” to “*yes, very much*”) to the questions.

Finally, acceptability of treatment will be assessed using the Treatment Acceptability/Adherence Scale (TAAS) [41]. The TAAS was developed to assess the acceptability of cognitive behavioral therapy (CBT) treatments for anxiety disorders, following the observation that despite such therapies having a strong evidence base, dropout rates may be high. It was selected for the trial given the view that EMDR can be seen as a form of CBT [42], the historical placement of PTSD among anxiety disorders within psychiatric classification schemes [31], and research showing the TAAS to have sound psychometric properties [41].

#### Secondary outcome measurements: effects of treatment on psychopathology

There are 10 secondary outcome measures (see Table 1). Firstly, the Posttraumatic Cognitions Inventory (PTCI) is a self-report measure assessing the level of negative thinking an individual has in relation to a specific trauma [43]. Subscales focus on self-blame, along with negative cognitions about the self and the world, which measure trauma-related cognitive distortions. The development and validation study published by the authors reports strong reliability coefficients and convergent validity with other PTSD measures [43].

The Trauma Symptom Inventory—second edition (TSI-2) [44] will be used to assess a broad range of post-trauma symptoms that can arise from acute, early life, and chronic traumatization. The TSI-2 is a 136-item self-report scale with factor analytically derived clinical subscales assessing: anxious arousal, depression, anger, intrusive experiences, defensive avoidance, dissociation, sexual disturbance, impaired self-reference, tension reduction behavior, somatic preoccupation, suicidality, and attachment insecurity. These are collapsed into the four additional factor analytically derived summary scales: self-disturbance, posttraumatic stress, externalization, and somatization. These four factors provide indices of broader complex posttraumatic difficulties beyond the DSM-5 construct of PSTD that may arise from early chronic interpersonal trauma [42]. The TSI also includes two validity scales (which evaluate the tendency to deny common symptoms and over-endorse uncommon symptoms), has extensive general population norms, and has strong psychometric properties [42].

The Beck Depression Inventory—second edition (BDI-II) [45] is a 21-item measure of cognitive, affective, and behavioral symptoms of depression. Items are rated from 0 to 3, with higher scores reflecting more depressive symptoms. The BDI-II is well established, both in international research and in clinical practice. Research supports the use of the BDI-II for screening for depressive disorders in adult mental health patients and is recognized for good internal consistency, retest reliability, and validity [46].

The World Health Organization Disability Assessment Schedule 2.0 (WHODAS 2.0) [47] is a self-report questionnaire that assesses limitations and disability in six domains: cognition; mobility; self-care; getting along with people; life activities, subdivided into household activities and work/school activities; and participation in society. The 12-item version will be used. For each item, respondents rate the level of difficulty experienced, in the prior month, on a 5-point scale from “*none*” to “*extreme or cannot do*”. The WHODAS 2.0 has been found to have good reliability and sensitivity to change across a range of physical and mental health conditions [48].

Psychotic symptoms will be measured by the PSYRATS delusions and hallucinations subscales [49]. The PSYRATS is an instrument that was developed to quantify the severity of delusions and hallucinations. It is commonly used in research studies and clinical settings focussing on people with psychotic illnesses.

Social functioning will be assessed using aspects of the Social Functioning Scale, a measure with sound psychometric properties, established acceptability for patients, and one of the more frequently used social functioning scales in SMI research [50, 51]. It has 79 items covering seven domains of functioning (social engagement/withdrawal; interpersonal communication; pro-social activities; recreation; independence–competence; independence–performance; employment/occupation), with standard scores for each subscale, and an “overall functioning” mean standard score is calculated. For the current trial, a number of questions will be omitted for those residing in secure settings as they are not relevant to that living situation.

Self-esteem will be measured by the Rosenberg Self Esteem Scale (RSES) [52]. This Likert scale has 10 items, each with 4-point scale response options from *strongly disagree* to *strongly agree*. It produces a total ranging from 0 to 30, with scores below 15 considered to reflect low self-esteem. The RSES is likely the most commonly used self-esteem measure in psychology research, and has shown satisfactory validity, reliability, and floor and ceiling effects [53].

To promote participant retention and adherence, we will present information about the study to health professional and patient groups before and during the study, to promote and sustain interest in the trial. We will enlist key worker (case manager) support to remind participants to attend treatment sessions and the follow-up assessments. The therapist and assessors will travel to the site where the participant is residing, to reduce logistical barriers to attending. The EMDR therapist will complete a record of attendance.

Randomized participants who discontinue treatment will have T1 and T2 assessments completed if possible. Once a participant is enrolled, the investigators will make every reasonable effort to follow them up for the entire study period.

#### Subjective experiences of EMDR: qualitative data collection

We are also interested in the subjective experiences of those experiencing PTSD and receiving EMDR treatment, and so this study also contains a qualitative component. We will use in-depth, semi-structured, audio-recorded interviews to explore people’s subjective experiences using a grounded theory approach. Interviews will occur with approximately 10 service users recruited sequentially (until data saturation is reached; *n* + 1) who have undergone EMDR in this trial. Due to the grounded theory study methodology, although there are broad themes and probes, the interviewer will adapt questioning to explore themes raised by the participant rather than sticking rigidly to the initial schedule.

### Sample size calculations

The power is based on information from investigations in related, but not identical, clinical groups to those planned to participate in this trial.

In an RCT investigating EMDR in mental health outpatients with psychosis and PTSD [24], the post-treatment CAPS score for EMDR was 40.3 (*n* = 55), compared with 56.5 (*n* = 47) for the waiting list, a difference of 16 points. The baseline mean (SD) CAPS score for these trial participants was 69.9 (16.2).

A 15-point change in CAPS total severity score has been proposed as a marker of clinically significant change [32], and so we feel that a difference of 16 points on the CAPS is clinically significant, and is likely to represent the difference between severe and moderate symptoms, or moderate and mild (subthreshold) symptoms.

Fifteen participants per arm are needed to detect a change of 16 points with an SD of 16 points using a *t* test and based on equal proportions of participants randomized to each group with a two-sided type I error rate of 0.05 and power of 0.8.

We estimate that attrition could be as much as 30%, although this is likely to be a conservative estimate as a meta-analysis reports that the dropout rate for trauma-focused interventions is around 18% and our previous research in this forensic patient group [29, 54, 55] has found participants to be well engaged with low dropout rates (< 15%). A conservative estimate of dropout is reasonable because the studies in the meta-analysis were over shorter timeframes, and we anticipate that some participants may have been discharged, changed address, or otherwise been lost to follow up at the 6-month mark. Based on the 30% dropout rate, the trial would need to recruit 23 participants per arm.

An important consideration is that we are uncertain whether the SD of the outcome variable will be as previously reported. To this end, we will initially plan to recruit 2 × 8 participants in order to estimate the SD, as the number of participants gives reasonable precision for this estimation. This will also enable us to determine preliminary efficacy, safety, and acceptability data, before further recruiting takes place.

To achieve participant enrolment we will work closely with clinical teams to facilitate targeted enrolment. The Central Regional Forensic Service covers five prisons. We plan to start the trial at two of these sites, selected for practical and representative purposes (one male and one female prison, close to the research center), but if we cannot achieve adequate enrolment at these initial sites, we will add further sites.

### Withdrawal of participants from treatment

Participants may withdraw from the study for any reason or at any time. The investigators may also withdraw participants from the study if there are concerns about their safety and/or if they are unwilling or unable to comply with study procedures. The criteria for discontinuing EMDR treatment include the following: the participant does not attend for more than three consecutive treatments or for more than four treatments in total; serious adverse events (defined in the next section); participant request; and Data Monitoring Committee recommendation (described in the next section). As already described, we will analyze participants using intention-to-treat methods.

### Trial monitoring and oversight

The risks of the study are thought to be low based on previous research, which has found EMDR to be safe, effective, and well tolerated in people with serious mental illness. However, the population is vulnerable so there are a number of safeguards in place. A Data Monitoring Committee (DMC) has been set up as an advisory body responsible for monitoring emerging safety and efficacy data, reviewing trial conduct and making recommendations. The DMC is independent of the study organizers and funders. Its membership is multidisciplinary and comprises the Clinical Director (Chair) and Operations Manager from the Central Regional Forensic and Rehabilitation Service, a senior psychologist not related to the study, and the principal investigator. During the recruitment period, interim analyses will be supplied in strict confidence to the DMC, together with any analyses the DMC may request.

The base rate of serious events in this population is much higher than in the general population, and it may be difficult to establish whether such events are related or unrelated to therapy. All serious adverse outcomes will be reported immediately to the DMC. We have predetermined that any serious event such as a serious deliberate self-harm attempt (requiring medical attention), an assault on another person (requiring medical attention), or a return to an environment of high security will be a reason for the participant being withdrawn from the study. Any other significant adverse effects will be discussed on a case-by-case basis. The DMC and research team will formally review the feasibility and safety of the study after data have been collected on 10 participants, before further participants are recruited.

The terms of reference of the DMC are available on request from the principal investigator.

In the unlikely event that someone is harmed in the course of the trial, they would be eligible under New Zealand law to apply for compensation from the Accident Compensation Corporation. This is explained in the informed consent process.

### Data management

Each participant will be allocated a unique participant number. Data will be coded and entered into an Excel spreadsheet in de-identified form. Data will be double checked by a secondary data analyst. Data will be stored securely in a locked cabinet for 10 years, or in a password-protected secure computer file. Data will be destroyed after 10 years.

All principal investigators will be given access to the cleaned and password-protected data set.

### Quantitative data analysis

Data analyses will be performed by a biostatistician blinded to treatment allocation using intention-to-treat and per-protocol analyses. The per-protocol analysis group refers to those participants who completed the treatment as originally allocated. We have defined this as participants who undergo at least six of the maximum nine total number of EMDR treatment sessions or who fulfill the criteria for early completion.

Baseline differences in demographic and clinical characteristics between the EMDR and control group will be analyzed using chi-squared tests, *t* tests, and analysis of variance. Descriptive statistics will provide data summaries. Continuous variables will be analyzed on an intention-to-treat basis with linear mixed models. For the PSYRATS data, which is usually not normally distributed because of a disproportionate number of zero scores, differences will be analyzed using the Wilcoxon signed-rank test and the Mann–Whitney *U* test.

Baseline scores will be included as covariates, time as a categorical variable, and treatment condition as a fixed effect. The intercept will be treated as a random effect. Dichotomous outcomes will be analyzed with logistic generalized estimating equation analyses with exchangeable correlation structure.

Effects will be calculated for post treatment and 6-month follow-up using interaction effects. Analyses of completers and intent-to-treat analyses with the last observation carried forward (with missing data on loss of diagnosis conservatively replaced with a negative value, i.e., no loss of diagnosis) will be performed to test the robustness of the findings.

The number needed to treat will be calculated to determine the number of participants who needed to be treated to make one more patient lose the PTSD diagnosis or achieve full remission compared with the control condition.

All tests will be two-sided and *p* < 0.05 will be considered statistically significant. We will use multiple imputations for missing data.

A biostatistician is part of the research team.

For the qualitative data, thematic analysis will be undertaken using NVivo software (QSR International). Two researchers will independently read and code the transcripts, the codes will be examined, and by an iterative process the codes will be condensed into groups that capture similar themes, each with a number of subthemes. These themes and subthemes will be considered by the research team, to validate the plausibility of the themes. To achieve saturation of the themes, the researchers will move back and forth between data collection and analysis, ensuring the fit between the data, and the conceptual work of analysis and interpretation. Work completed early in the study will inform subsequent recruitment, data collection, and analysis.

### Publication of results

We will make every endeavor to keep to a minimum the interval between the completion of the data collection and the release of the study results. We expect to take about 4 months to compile the final results for an appropriate journal. For publications, authorship eligibility will be determined according to the International Committee of Medical Journal Editors authorship criteria [56], and assessed by the Trial Steering Committee. We will not use professional medical writers. The study results will be released to the participating sites, the referring physicians, the patients, and the general mental health community. The study sponsor will not impose restrictions on publication.

## Discussion

A substantial proportion of forensic and rehabilitation mental health clients in long-stay settings experience PTSD. The potential for symptoms of trauma to impede recovery and contribute to deterioration in well-being is well established in the literature. This study aims to explore an important gap in the literature. While EMDR is a well-established treatment for PTSD in the general population, and there is emerging evidence for its effectiveness for people with SMI, there are no RCTs assessing the use of this intervention for PSTD with a forensic mental health population.

If efficacy can be demonstrated, the results of this trial will enhance availability and foster dissemination of evidence-based treatment of PTSD within the forensic and rehabilitation services. Ultimately, the use of EMDR may result in greater well-being and quality of life, together with a reduction in reoffending, for those individuals with PTSD in forensics settings.

## Trial status

This trial is currently underway. Recruitment of participants started on 25 May 2018. It is anticipated the last patient will be enrolled on 13 November 2020 and the last data will be collected by 17 July 2021. The study was registered prospectively with the Australian New Zealand Clinical Trial Network. This protocol is based on version 3.0 of 7 September 2019. The items from the World Health Organization Trial Registration Data set are summarized in Table 2.

## Supplementary information

**Additional file 1.** SPIRIT 2013 Checklist: Recommended items to address in a clinical trial protocol and related documents.

## Data Availability

The datasets generated and analyzed during the current study are not publicly available due to the sensitivity of some of the material but will be available from the corresponding author on reasonable request.

## Abbreviations

AEQ: Adverse Events Questionnaire
BDI-II: Beck Depression Inventory—second edition
CAPS: Clinician Administered PTSD Scale
CONSORT: Consolidated Standards of Reporting of Trials
DSM-5: *Diagnostic and Statistical Manual of Mental Disorders*, fifth edition
EMDR: Eye movement desensitization and reprocessing
NC: Negative cognition
PC: Positive cognition
PSS-SR: Posttraumatic Stress Disorder Symptom Scale—Self-Report
PSYRATS: Psychotic Symptom Rating Scales
PTSD: Posttraumatic stress disorder
RSES: Rosenberg Self-Esteem Scale
SFS: Social Functioning Scale
SMI: Serious mental illness
SUD: Subjective Units of Distress
TAAS: Treatment Acceptability/Adherence Scale
TSI-2: Trauma Symptom Inventory—second edition
TSQ: Trauma Screening Questionnaire
VOC: Validity of Cognition
WHODAS: World Health Organization Disability Assessment Scale

## Footnotes

1 Māori are the indigenous people of New Zealand.
